# The Nutri-Exposome Intelligence Framework: Integrating Multi-Omics, Machine Learning, and Digital Nutrition for Precision Chronic Disease Prevention

**DOI:** 10.3390/nu18111826

**Published:** 2026-06-05

**Authors:** Mia Yang Ang, Siew Woh Choo

**Affiliations:** 1 Department of Biomedical Sciences, Jeffrey Cheah Sunway Medical School, Faculty of Medical and Life Sciences, Sunway University, Bandar Sunway, Petaling Jaya 47500, Selangor, Malaysia; 2 Sunway Microbiome Centre, Faculty of Medical and Life Sciences, Sunway University, Bandar Sunway, Petaling Jaya 47500, Selangor, Malaysia; 3 Zhejiang-Malaysia Joint Laboratory for Rare Medicinal Resources, Wenzhou-Kean University, 88 Daxue Road, Ouhai, Wenzhou 325060, China; 4 College of Science, Mathematics and Technology, Wenzhou-Kean University, 88 Daxue Road, Ouhai, Wenzhou 325060, China; 5 International Frontier Interdisciplinary Research Institute (IFIRI), Wenzhou-Kean University, 88 Daxue Road, Ouhai, Wenzhou 325060, China; 6 Dorothy and George Hennings College of Science, Mathematics and Technology, Kean University, 1000 Morris Ave, Union, NJ 07083, USA

**Keywords:** precision nutrition, nutri-exposome, machine learning, multi-omics, gut microbiome, digital nutrition, exposomics, chronic disease prevention, personalized dietary intervention

## Abstract

**Background/Objectives:** Precision nutrition is moving beyond population-based guidance and isolated gene–diet interactions toward integrative models of dietary response. However, current approaches remain fragmented across nutrigenomics, microbiome research, multi-omics profiling, digital health, and machine learning. This review proposes the Nutri-Exposome Intelligence Framework as a conceptual, data science-driven model for integrating cumulative dietary, environmental, microbial, molecular, clinical, and digital exposures for precision chronic disease prevention. **Methods:** This conceptual review synthesizes the literature on precision nutrition, nutrigenetics, nutrigenomics, exposomics, gut microbiome research, multi-omics integration, wearable and biomarker-based monitoring, and machine learning in nutrition studies. Evidence was organized into a framework linking exposure assessment, host susceptibility, microbiome-mediated biotransformation, molecular response profiling, computational modelling, personalized intervention, and longitudinal feedback. **Results:** The proposed framework consists of seven interconnected layers: diet, environment, and lifestyle exposures; host genome and microbiome; multi-omics molecular responses; machine learning-based integration; risk prediction and responder stratification; personalized dietary intervention; and wearable and biomarker-based feedback. It positions the nutri-exposome as a cumulative exposure–response system and highlights how machine learning can support data harmonization, feature engineering, predictive modelling, responder classification, explainable interpretation, and adaptive refinement of dietary recommendations. Key applications include obesity, type 2 diabetes, cardiovascular disease, metabolic dysfunction-associated steatotic liver disease, cardiovascular–kidney–metabolic syndrome, and broader cardiometabolic prevention. **Conclusions:** Nutri-exposome intelligence offers a structured pathway for transforming complex nutrition data into predictive, explainable, and adaptive precision nutrition strategies. Implementation will require longitudinal and multi-ethnic cohorts, standardized metadata, causal validation, interpretable machine learning, ethical governance, and equitable access to support responsible clinical and public health translation globally.

## 1. Introduction

Precision nutrition is increasingly shaped by the convergence of dietary assessment, molecular profiling, microbiome research, digital health monitoring, and computational modelling [1]. These developments create an opportunity to move beyond generalized dietary advice toward more individualized and biologically informed prevention strategies [2].

### 1.1. From Population-Based Nutrition to Precision Nutrition

Population-based dietary guidelines remain essential for public health because they provide broadly applicable recommendations to reduce diet-related disease risk [3]. These guidelines have supported health promotion by encouraging balanced dietary patterns, adequate nutrient intake, increased consumption of fruits, vegetables, whole grains, and dietary fiber, and reduced intake of excess sugar, salt, and saturated fat [4]. However, such guidelines are not designed to explain why individuals exposed to similar diets may show markedly different metabolic, inflammatory, microbial, and clinical responses. Differences in postprandial glucose response, lipid metabolism, body weight regulation, inflammatory status, gut microbiome composition, and chronic disease susceptibility highlight the biological variability underlying dietary response.

Precision nutrition has emerged to address this limitation by tailoring dietary strategies according to individual biological, metabolic, behavioral, and environmental characteristics [5]. Rather than asking only which diet is generally healthy, precision nutrition asks which dietary intervention is most suitable for a specific individual or subgroup under defined biological and lifestyle conditions [6]. This approach is particularly relevant to chronic disease prevention, where obesity, type 2 diabetes, cardiovascular disease, metabolic dysfunction-associated steatotic liver disease, and cardiometabolic disorders develop through long-term interactions among diet, host biology, microbiome function, lifestyle, and environmental exposure.

### 1.2. Beyond Gene-Centered Nutrigenomics: Toward the Nutri-Exposome

Early precision nutrition research was strongly influenced by nutrigenetics and nutrigenomics [7]. Nutrigenetics examines how inherited genetic variation affects individual responses to nutrients, while nutrigenomics investigates how nutrients and dietary bioactive compounds regulate gene expression and downstream molecular pathways [8]. These fields have provided important insights into gene–diet interactions involving lipid metabolism, glucose regulation, methylation pathways, inflammation, appetite control, and obesity susceptibility. Variants in genes such as *APOE*, *FTO*, *MTHFR*, *APOA5*, and *TCF7L2* have been associated with differences in nutrient metabolism, cardiometabolic risk, or dietary intervention response.

Despite these contributions, gene-centered nutrigenomics has important limitations [9]. Genetic variants are relatively stable, but dietary responses are dynamic, cumulative, and context dependent [10]. The same genetic background may lead to different phenotypic outcomes depending on age, sex, ancestry, baseline metabolic status, gut microbiome composition, medication use, physical activity, sleep quality, stress, socioeconomic conditions, and environmental exposure. The exposome provides a useful conceptual foundation for expanding this perspective. In nutritional science, the nutri-exposome can be understood as the cumulative and dynamic set of dietary, nutritional, microbial, environmental, behavioral, and metabolic exposures that interact with host biology across the life course to influence health and disease risk. This broader framing positions the nutri-exposome as a life-course exposure–response model for understanding diet-related chronic disease risk.

### 1.3. Data Science and Machine Learning as Enablers of Nutri-Exposome Intelligence

Advances in high-throughput technologies now make it possible to measure multiple layers of the nutri-exposome [11]. Genomics provides information on inherited susceptibility, epigenomics captures regulatory memory of exposure, transcriptomics reflects active gene expression responses, proteomics measures functional protein-level changes, metabolomics and lipidomics reveal biochemical consequences of diet and metabolism, and microbiomics characterizes microbial communities and functions that transform dietary substrates into bioactive metabolites [12]. When combined with dietary assessment, clinical biomarkers, food environment data, wearable sensors, and continuous glucose monitoring, these data layers provide a multidimensional view of individual dietary response.

However, nutri-exposome data are high-dimensional, heterogeneous, longitudinal, and often incomplete [13]. Conventional analytical approaches may be insufficient to identify meaningful patterns across diverse dietary, molecular, microbial, clinical, and digital data sources [14]. Data science and machine learning are therefore becoming central to the next phase of precision nutrition. Machine learning can support dietary pattern recognition, feature selection, biomarker discovery, disease-risk prediction, responder stratification, microbiome-metabolite modelling, and multi-omics integration. In this review, we propose the Nutri-Exposome Intelligence Framework as a data science-driven model that integrates external dietary and environmental exposures, host biological susceptibility, microbiome-mediated biotransformation, multi-omics molecular responses, machine learning-based interpretation, precision nutrition intervention, and real-time feedback monitoring for chronic disease prevention.

The specific contribution of the Nutri-Exposome Intelligence Framework is that it organizes currently fragmented areas of precision nutrition into a single exposure–response–feedback architecture [15]. Existing precision nutrition models commonly emphasize genetic variation, microbiome composition, metabolic profiling, dietary assessment, or digital monitoring as separate domains [16]. In contrast, the proposed framework explicitly links external dietary and environmental exposures, host susceptibility, microbiome-mediated biotransformation, multi-omics molecular response, machine learning-based interpretation, personalized intervention, and longitudinal feedback as interconnected layers of one adaptive prevention system. Thus, the framework is not intended to replace existing nutrigenomics, exposomics, or multi-omics models, but to provide an implementation-oriented structure for integrating them in chronic disease prevention research.

### 1.4. Review Approach and Literature Selection

A structured conceptual review approach was used to develop and contextualize the proposed framework, rather than to conduct a systematic review or meta-analysis [17]. The literature search was designed to identify key conceptual, methodological, and translational studies relevant to precision nutrition, exposomics, nutrigenomics, gut microbiome research, multi-omics integration, wearable and biomarker-based nutrition monitoring, and machine learning in nutrition studies [18]. The search strategy was informed by PRISMA principles for transparent reporting, although formal risk-of-bias assessment and quantitative synthesis were not performed because the objective was to develop and contextualize a conceptual framework rather than estimate pooled intervention effects.

Literature was identified through PubMed, Scopus, Web of Science, Google Scholar, and reference chaining. Search terms included combinations of “precision nutrition”, “personalized nutrition”, “nutrigenomics”, “nutrigenetics”, “exposome”, “nutri-exposome”, “gut microbiome”, “multi-omics”, “metabolomics”, “continuous glucose monitoring”, “wearable sensors”, “machine learning”, “artificial intelligence”, “responder stratification”, “dietary intervention”, “cardiometabolic disease”, “type 2 diabetes”, “obesity”, “MASLD”, and “cardiovascular-kidney-metabolic syndrome”. Priority was given to peer-reviewed reviews, consensus papers, major cohort or intervention studies, methodological articles, and recent machine learning applications in nutrition and microbiome research.

## 2. Conceptual Foundations of the Nutri-Exposome Intelligence Framework

The nutri-exposome provides a broader way to understand dietary response by placing nutrition within a cumulative exposure–response system [19]. Rather than treating diet, host biology, microbiome activity, molecular regulation, and lifestyle as separate domains, the framework connects them as interacting layers that shape chronic disease risk over time [20].

### 2.1. Defining the Nutri-Exposome

The nutri-exposome refers to the cumulative and dynamic set of dietary, nutritional, microbial, environmental, behavioral, and metabolic exposures that interact with host biology across the life course [21]. It extends the concept of the exposome into nutritional science by placing diet at the center of exposure–response biology [22]. In this framework, food is not viewed only as a source of calories or nutrients, but as a complex exposure system composed of macronutrients, micronutrients, bioactive compounds, additives, contaminants, food processing signatures, dietary patterns, meal timing, and culturally shaped eating behaviors. These exposures interact continuously with host genetics, epigenetic regulation, microbial metabolism, immune signaling, and clinical physiology.

This concept is broader than conventional nutrigenomics [23]. Nutrigenomics commonly focuses on how nutrients influence gene expression, while nutrigenetics focuses on how genetic variants modify nutrient response [7]. The nutri-exposome expands this view by asking how cumulative dietary and environment-related exposures are biologically transformed, molecularly embedded, and clinically expressed over time. This is particularly important for chronic disease prevention because conditions such as obesity, type 2 diabetes, cardiovascular disease, metabolic dysfunction-associated steatotic liver disease, and cardiometabolic syndrome are not caused by single nutrients or isolated genetic variants. Instead, they emerge from repeated and interacting exposure patterns that shape metabolic, inflammatory, microbial, and molecular trajectories. The conceptual distinction between nutrigenetics, nutrigenomics, exposomics, precision nutrition, and the proposed Nutri-Exposome Intelligence Framework is summarized in Table 1.

### 2.2. From Multi-Omics Profiling to Nutri-Exposome Intelligence

Multi-omics technologies provide the biological measurement layer required to study the nutri-exposome [35]. By capturing genomic susceptibility, epigenetic regulation, transcriptomic activity, protein-level function, metabolic state, lipid profiles, microbial composition, and immune response, these platforms allow dietary exposures to be linked to molecular and physiological changes [7]. These omics layers should not be interpreted as isolated datasets, but as interconnected components of a broader exposure–response system. For example, dietary patterns may shape gut microbial metabolism, which may subsequently influence metabolite availability, immune signalling, lipid regulation, and chronic disease risk.

However, multi-omics profiling alone does not automatically generate clinically actionable knowledge [36]. The value of nutri-exposome research depends on the ability to organize diverse data layers into interpretable models that explain how exposures are transformed into measurable biological responses [13]. In this context, nutri-exposome intelligence refers to the use of data science and machine learning to integrate dietary, environmental, microbial, molecular, clinical, and digital data into predictive, explainable, and adaptive models of dietary response. By linking biological measurement with computational interpretation, this approach provides a pathway for translating complex nutrition data into precision prevention strategies.

### 2.3. Core Structure of the Nutri-Exposome Intelligence Framework

The Nutri-Exposome Intelligence Framework is proposed as a seven-layer model for precision nutrition and chronic disease prevention [37]. The first layer is the external exposure layer, which includes diet, food environment, lifestyle, pollutants, contaminants, medication use, socioeconomic context, and behavioral patterns [38]. The second layer is host biological susceptibility, including genomics, polygenic risk, age, sex, ancestry, epigenetic background, and baseline metabolic health. The third layer is microbiome-mediated biotransformation, where dietary substrates are converted into microbial metabolites such as short-chain fatty acids, bile acid derivatives, indoles, phenolic metabolites, and trimethylamine-related compounds.

**Figure 1:**
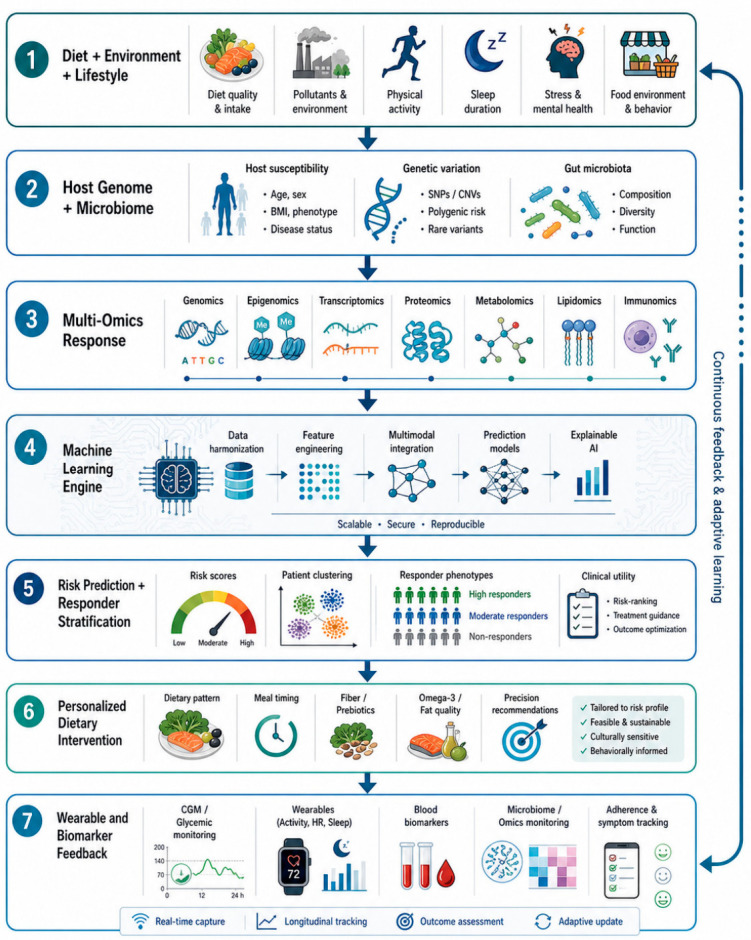
The Nutri-Exposome Intelligence Framework for precision chronic disease prevention. The framework integrates seven interconnected layers: diet, environment, and lifestyle exposures; host genome and microbiome; multi-omics molecular responses; machine learning-based data integration; risk prediction and responder stratification; personalized dietary intervention; and wearable and biomarker-based feedback. The circular feedback loop indicates continuous monitoring and adaptive refinement of dietary recommendations based on longitudinal biological and behavioral response data.

The fourth layer is the multi-omics molecular response layer, which captures how exposures are translated into gene regulation, protein activity, metabolic signatures, immune responses, and disease-associated molecular phenotypes [39]. The fifth layer is the machine learning integration layer, where computational models identify patterns, select predictive features, classify responder subgroups, estimate disease risk, and support explainable interpretation [40]. The sixth layer is the precision intervention layer, where dietary strategies are tailored according to individual or subgroup profiles. The seventh layer is real-time feedback monitoring, using tools such as continuous glucose monitoring, wearable sensors, mobile dietary records, repeated biomarkers, and n-of-1 intervention designs. Together, these layers form a feedback system in which dietary recommendations can be continuously refined based on measured biological response. The overall structure of the proposed Nutri-Exposome Intelligence Framework is illustrated in Figure 1, highlighting the sequential integration of dietary, environmental, host, microbiome, multi-omics, machine learning, intervention, and feedback layers.

## 3. Data Sources and Measurement Layers for Nutri-Exposome Modelling

Nutri-exposome modelling depends on data that capture both external exposures and internal biological responses [41]. Dietary intake, environmental context, host susceptibility, microbiome function, molecular profiles, clinical phenotypes, and longitudinal feedback each contribute different information to the overall exposure–response picture [21].

### 3.1. Dietary, Environmental, and Digital Phenotyping Data

Accurate characterization of dietary exposure is the foundation of nutri-exposome modelling [42]. Traditional approaches, including food frequency questionnaires, 24 h dietary recalls, food diaries, and diet history interviews, remain widely used because they provide structured information on nutrient intake and dietary patterns [43]. However, these methods are limited by recall bias, underreporting, inconsistent portion-size estimation, and difficulty capturing day-to-day dietary variability. In the context of nutri-exposome intelligence, dietary data should extend beyond nutrient quantification to include meal timing, food processing level, cooking methods, food additives, dietary supplements, cultural eating patterns, and the frequency of ultra-processed food intake.

Environmental and digital phenotyping data add further depth to dietary exposure assessment [43]. The food environment, socioeconomic context, physical activity, sleep quality, medication use, stress, smoking, alcohol intake, and exposure to pollutants or food contaminants may all modify the biological effects of diet [38]. Wearable sensors, mobile dietary applications, continuous glucose monitoring, smart scales, sleep trackers, and activity monitors can provide longitudinal and real-time data that complement conventional dietary assessment. These data streams are important because they allow nutrition studies to move from static exposure measurement toward dynamic monitoring of how individuals respond to diet under real-world conditions.

### 3.2. Host, Microbiome, and Multi-Omics Data

Host biological data provide the internal context in which dietary exposures are interpreted [44]. Genomic information can identify inherited susceptibility to nutrient-related phenotypes, while polygenic risk scores may support disease-risk stratification when interpreted alongside clinical and lifestyle factors [7]. Epigenomic data provide insight into regulatory changes associated with long-term exposure, including DNA methylation, histone modification, and non-coding RNA regulation. Transcriptomic, proteomic, metabolomic, and lipidomic data further capture active molecular responses to dietary intake, microbial metabolism, inflammation, oxidative stress, and cardiometabolic dysfunction. Together, these omics layers provide a more complete view of how external exposures are translated into biological response.

The gut microbiome is a particularly important component of nutri-exposome modelling because it functions as a metabolic interface between diet and host physiology [45]. Dietary substrates are transformed by microbial communities into bioactive metabolites, including short-chain fatty acids, secondary bile acids, indoles, phenolic metabolites, and trimethylamine-related compounds [21]. These metabolites can influence host energy metabolism, immune regulation, epithelial barrier function, lipid metabolism, and inflammatory pathways. This integrated perspective allows researchers to examine not only dietary intake but also the biological transformation of dietary compounds and their downstream effects on chronic disease risk.

### 3.3. Clinical Phenotypes and Longitudinal Feedback Data

Clinical phenotypes are essential for linking nutri-exposome profiles to measurable health outcomes [46]. Anthropometric measures such as body mass index, waist circumference, body composition, and blood pressure provide accessible indicators of metabolic health, while biochemical markers such as fasting glucose, insulin, HbA1c, lipid profile, liver enzymes, inflammatory markers, and renal function markers offer more detailed information on disease risk and intervention response [43]. For chronic disease prevention, these phenotypes should not be interpreted as isolated endpoints, but as measurable outputs of long-term interactions among diet, host biology, microbiome function, lifestyle, and environmental exposure.

Longitudinal feedback data add temporal resolution to nutri-exposome modelling [47]. Repeated measurements of glucose variability, lipid response, body weight, metabolite signatures, microbiome composition, dietary adherence, physical activity, and sleep can help distinguish transient dietary responses from more stable biological patterns [21]. These repeated measurements are also important for n-of-1 nutrition studies, where individual-level response trajectories are used to evaluate the effects of dietary intervention over time. The major data sources, measurement layers, analytical roles, and implementation challenges relevant to nutri-exposome modelling are summarized in Table 2.

### 3.4. Available Data Resources for Nutri-Exposome Intelligence Studies

A practical limitation of nutri-exposome intelligence is that few studies contain all the data layers required for comprehensive modelling [60]. Therefore, implementation should begin with available datasets and expand progressively as new cohorts, omics layers, and digital monitoring streams become available [61]. Public and controlled-access datasets can support hypothesis generation, method development, external validation, and benchmarking, whereas prospective and private datasets are often required for real-time intervention and adaptive feedback models. Representative dataset categories are summarized in Table 3.

## 4. Machine Learning Strategies for Nutri-Exposome Integration

The complexity of nutri-exposome data creates both an opportunity and a challenge for computational analysis [75]. Machine learning can help organize heterogeneous data, identify response patterns, support risk prediction, and generate interpretable features, but its value depends on careful preprocessing, validation, and biological interpretation [38].

### 4.1. Data Harmonization and Feature Engineering

Nutri-exposome modelling requires the integration of highly heterogeneous data sources, including dietary records, clinical phenotypes, microbiome profiles, metabolomics, genomics, wearable sensor outputs, and environmental exposure data [76]. These datasets differ in scale, structure, resolution, sampling frequency, and biological meaning [60]. For example, dietary intake may be recorded as daily food items or nutrient estimates, while continuous glucose monitoring produces dense time-series data and multi-omics platforms generate high-dimensional molecular features. Before machine learning can be applied, these data must be harmonized through preprocessing, normalization, missing-value handling, metadata standardization, and correction for technical variation such as batch effects.

Feature engineering is equally important because raw nutrition and omics data are not always directly interpretable [77]. Dietary variables may be transformed into nutrient densities, dietary pattern scores, food processing categories, meal-timing indicators, or exposure indices [5]. Microbiome data may be summarized as taxonomic abundance, functional pathways, diversity measures, or metabolite-producing capacity. Wearable and continuous glucose monitoring data can be converted into features such as glucose variability, postprandial response, sleep duration, physical activity intensity, and circadian regularity. These engineered features provide the input layer for machine learning models and help connect complex nutri-exposome data to biologically meaningful patterns.

### 4.2. Predictive Modelling and Responder Stratification

Machine learning can support precision nutrition by identifying patterns that are difficult to detect using conventional statistical approaches [40]. Supervised learning methods, such as random forest, support vector machines, gradient boosting, and regularized regression, can be used to predict disease risk, dietary response, biomarker changes, or intervention outcomes [1]. In chronic disease prevention, these models may help estimate the likelihood of developing obesity, type 2 diabetes, cardiovascular disease, metabolic dysfunction-associated steatotic liver disease, or broader cardiometabolic risk based on combined dietary, molecular, microbial, and clinical features.

Another important function of machine learning is responder stratification [78]. Individuals may respond differently to the same dietary intervention because of differences in host genetics, baseline metabolic status, microbiome function, lifestyle, and environmental exposure [7]. Unsupervised learning methods, including clustering, dimensionality reduction, and latent factor models, can identify subgroups with shared biological or dietary response profiles. These subgroups may include glucose-sensitive responders, lipid-sensitive responders, inflammation-dominant responders, microbiome-mediated responders, or individuals with poor metabolic flexibility. Such stratification allows precision nutrition to move beyond average treatment effects and toward targeted intervention design.

Several studies illustrate how machine learning has already been applied to precision nutrition, although clinical translation remains incomplete [79]. Zeevi et al. developed a model for predicting personalized postprandial glycemic responses by integrating dietary habits, anthropometrics, physical activity, blood parameters, and gut microbiota, followed by validation in an independent cohort [60]. PREDICT 1 extended this direction by examining inter-individual variation in postprandial triglyceride, glucose, and insulin responses using meal, microbiome, genetic, metabolomic, and contextual variables [58]. Food4Me provides a complementary example of personalized nutrition intervention testing, where different levels of dietary, phenotypic, and genotypic personalization were compared with standard dietary advice [80]. These studies show that machine learning can support response prediction and stratification, but they also demonstrate that clinical usefulness depends on external validation, feasibility, interpretability, and evidence that model-guided recommendations improve outcomes beyond standard care.

### 4.3. Explainable and Adaptive Machine Learning for Precision Nutrition

For nutri-exposome intelligence to be clinically useful, machine learning models must be interpretable, reproducible, and biologically plausible [81]. Black-box predictions are insufficient for nutrition practice because clinicians, dietitians, researchers, and patients need to understand why a specific dietary recommendation is generated [40]. Explainable artificial intelligence approaches can help identify which features contribute most strongly to a prediction, such as dietary fiber intake, glucose variability, microbial metabolite signatures, lipidomic markers, inflammatory proteins, or genetic risk scores. This interpretability is essential for linking computational outputs to nutritional mechanisms and for increasing trust in data-driven recommendations.

Adaptive machine learning further extends precision nutrition by allowing models to incorporate newly collected data rather than relying only on baseline measurements [82]. In the context of nutri-exposome integration, adaptive models may use repeated dietary records, wearable signals, continuous glucose monitoring, clinical biomarkers, and selected omics measurements to refine risk estimates and response classifications over time [1]. The goal is not only to improve predictive accuracy, but also to support biologically interpretable model updating as individual exposure and response profiles change. The main machine learning strategies relevant to nutri-exposome integration, together with their analytical roles, outputs, and implementation challenges, are summarized in Table 4.

### 4.4. Tiered Implementation of the Nutri-Exposome Intelligence Framework

**Figure 2:**
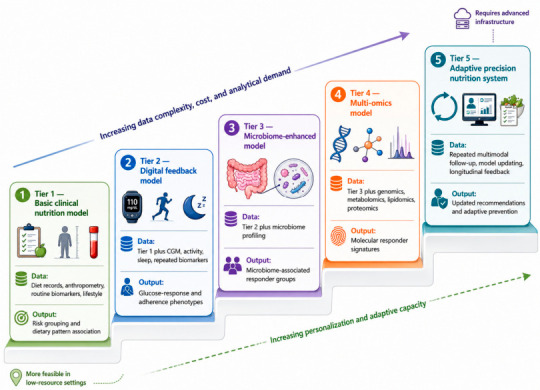
Tiered implementation model for nutri-exposome intelligence studies. The framework can be implemented progressively from basic clinical nutrition datasets to digital feedback, microbiome-enhanced, multi-omics, and adaptive precision nutrition systems, depending on data availability, infrastructure, cost, and analytical capacity.

The Nutri-Exposome Intelligence Framework should not be interpreted as requiring every study to collect all possible data layers [94]. In practice, implementation can follow a tiered design according to research objective, budget, infrastructure, and participant burden [38]. A minimum viable implementation may include dietary intake, anthropometry, routine clinical biomarkers, lifestyle data, and basic statistical or machine learning analysis. An intermediate implementation may add continuous glucose monitoring, wearable sensor data, repeated biomarkers, microbiome profiling, and more detailed dietary metadata. An advanced implementation may incorporate genomics, metabolomics, lipidomics, proteomics, environmental exposure measurements, repeated multi-omics profiling, and longitudinal adaptive modelling. This tiered structure allows the framework to guide both high-resource multi-omics cohorts and lower-resource studies that begin with clinically accessible data. The tiered implementation model is shown in Figure 2.

The analytical strategy should be matched to the implementation tier [95]. Tier 1 studies may use cross-sectional or short-intervention designs with regression, random forest, or gradient boosting approaches. Tier 2 studies may use short longitudinal or n-of-1 designs with time-series features, mixed models, or supervised machine learning. Tier 3 and Tier 4 studies require microbiome and multi-omics integration, where feature selection, pathway-based modelling, multi-view integration, and network-based approaches may be appropriate. Tier 5 represents the most advanced implementation level and is best suited to longitudinal cohorts or clinical implementation studies using adaptive machine learning, causal modelling, or privacy-preserving multi-site approaches.

## 5. Disease Applications of the Nutri-Exposome Intelligence Framework

Chronic diseases such as obesity, type 2 diabetes, cardiovascular disease, MASLD, and CKM syndrome develop through long-term interactions among diet, metabolism, inflammation, microbiome activity, lifestyle, and clinical vulnerability [96]. These conditions therefore provide useful examples for illustrating how nutri-exposome intelligence may support more targeted prevention strategies [13].

### 5.1. Obesity and Type 2 Diabetes

Obesity and type 2 diabetes are key target areas for nutri-exposome intelligence because both conditions are strongly influenced by long-term interactions among diet, genetic susceptibility, gut microbiome function, metabolic flexibility, physical activity, sleep, and environmental context [97]. Traditional approaches often classify risk using body mass index, fasting glucose, insulin resistance, or family history, but these markers may not fully capture the molecular heterogeneity underlying disease development [98]. Within the Nutri-Exposome Intelligence Framework, dietary intake, food processing level, meal timing, microbiome-derived metabolites, glucose variability, lipidomic signatures, inflammatory markers, and genetic risk can be integrated to identify individuals who are more likely to develop metabolic dysfunction or respond differently to dietary intervention.

Machine learning can support obesity and diabetes prevention by identifying responder subgroups and predicting individual responses to dietary patterns [99]. For example, some individuals may show exaggerated postprandial glucose responses to specific carbohydrate sources, while others may be more sensitive to dietary fat quality, low fiber intake, circadian eating disruption, or microbiome-mediated metabolic effects [21]. Integrating continuous glucose monitoring, microbiome profiles, metabolomics, and dietary records can help classify individuals into glucose-sensitive, insulin-resistant, inflammation-dominant, or microbiome-mediated response phenotypes. This allows dietary strategies to be refined beyond general calorie restriction toward more precise interventions involving carbohydrate quality, dietary fiber, protein distribution, meal timing, prebiotic intake, and long-term metabolic monitoring.

### 5.2. Cardiovascular and Cardiometabolic Disease

Cardiovascular disease and broader cardiometabolic risk are closely linked to dietary exposures, lipid metabolism, inflammation, endothelial function, oxidative stress, and host genetic susceptibility [100]. Conventional prevention strategies focus on established clinical markers such as blood pressure, low-density lipoprotein cholesterol, triglycerides, fasting glucose, and body weight [98]. Although these markers remain important, they do not fully explain why individuals differ in response to dietary fats, sodium, fiber, polyphenols, omega-3 fatty acids, or plant-based dietary patterns. A nutri-exposome approach expands cardiovascular nutrition research by integrating genetic variants, lipidomic profiles, inflammatory proteins, metabolomic markers, gut microbial metabolism, food environment, and lifestyle-derived digital data.

The microbiome is particularly relevant in cardiovascular and cardiometabolic disease because microbial metabolism can influence bile acid profiles, lipid handling, systemic inflammation, and production of metabolites such as trimethylamine-related compounds [101]. Machine learning models can integrate these microbial and molecular features with dietary exposure data to improve risk prediction and identify intervention targets [21]. For instance, individuals with lipid-sensitive profiles may benefit from targeted modification of saturated fat quality, dietary fiber, and plant sterol intake, whereas inflammation-dominant profiles may require broader lifestyle-oriented strategies that emphasize anti-inflammatory dietary patterns, weight management, sleep optimization, and physical activity. In this context, nutri-exposome intelligence provides a pathway for moving from broad cardiovascular dietary advice toward biologically stratified prevention.

### 5.3. MASLD, CKM Syndrome, and Multi-System Chronic Disease Prevention

Metabolic dysfunction-associated steatotic liver disease, cardiovascular–kidney–metabolic syndrome, and related multi-system chronic diseases highlight the need for integrated nutritional frameworks [98]. These conditions involve overlapping disturbances in hepatic lipid accumulation, insulin resistance, systemic inflammation, renal stress, vascular dysfunction, adipose tissue biology, and gut–liver axis signaling [21]. A single biomarker or single-omics approach is unlikely to capture this complexity. The Nutri-Exposome Intelligence Framework is therefore well suited for these conditions because it allows dietary exposure, host susceptibility, microbiome activity, metabolomics, lipidomics, inflammatory markers, and longitudinal clinical phenotypes to be interpreted as interacting components of the same disease network.

For MASLD and CKM-related risk, machine learning can be used to identify multi-system risk signatures and predict which individuals are more likely to benefit from specific dietary interventions [102]. These may include energy restriction, Mediterranean-style dietary patterns, reduced intake of ultra-processed foods, improved carbohydrate quality, increased dietary fiber, targeted protein distribution, and microbiome-modulating strategies [21]. Repeated monitoring of liver enzymes, lipid profiles, glucose variability, renal markers, body composition, inflammatory biomarkers, and metabolomic signatures can support adaptive intervention refinement. The disease-specific applications of the Nutri-Exposome Intelligence Framework, including relevant exposure layers, molecular mediators, machine learning tasks, and potential precision nutrition outputs, are summarized in Table 5.

## 6. Translational Roadmap for Implementing Nutri-Exposome Intelligence

**Figure 3:**
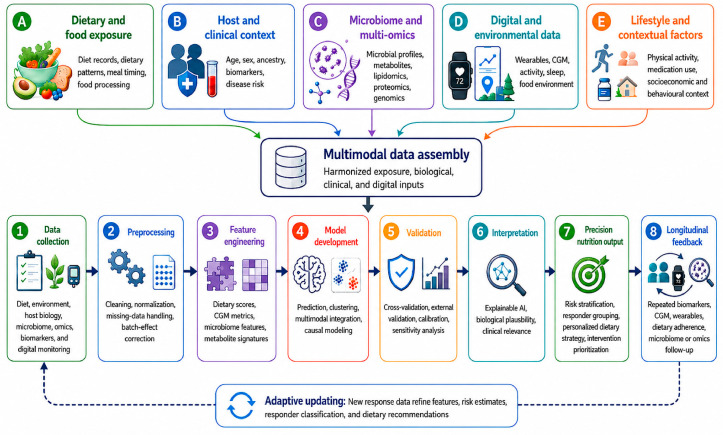
Data-to-model workflow for implementing nutri-exposome intelligence. The workflow shows how multimodal nutrition-related data can be processed, modelled, interpreted, and refined through longitudinal feedback to support adaptive precision nutrition.

Translating nutri-exposome intelligence into real-world research or clinical implementation requires more than assembling multiple data layers [112]. It requires a structured workflow that connects data collection, preprocessing, feature engineering, multimodal integration, model development, validation, interpretation, intervention design, and longitudinal feedback [38]. Figure 3 summarizes this data-to-model workflow and illustrates how heterogeneous dietary, environmental, host, microbiome, multi-omics, clinical, and digital data may be converted into interpretable outputs for precision nutrition research.

### 6.1. From Baseline Profiling to Personalized Risk Stratification

The first step in implementing the Nutri-Exposome Intelligence Framework is comprehensive baseline profiling [113]. This process should integrate dietary intake, clinical history, anthropometric measurements, biochemical markers, medication use, lifestyle factors, environmental exposures, and, where feasible, genomic and microbiome data [21]. Rather than relying on a single marker such as body mass index, fasting glucose, or genetic risk alone, baseline profiling should capture the broader biological and exposure context that shapes dietary response. This is particularly important for chronic disease prevention because individuals with similar clinical profiles may have different molecular drivers of risk, including insulin resistance, lipid dysregulation, inflammatory activation, microbial imbalance, or impaired metabolic flexibility.

Machine learning can then be used to support personalized risk stratification by identifying patterns across these heterogeneous data layers [114]. Individuals may be classified into subgroups based on predicted dietary response, disease risk trajectory, or dominant biological mechanism [40]. For example, one subgroup may show strong postprandial glucose variability, another may show lipid-sensitive cardiometabolic risk, while another may display inflammation-dominant or microbiome-mediated metabolic dysfunction. Such stratification allows precision nutrition to move beyond generalized dietary advice and toward targeted intervention strategies that are matched to the individual’s biological and exposure profiles.

### 6.2. Designing Adaptive Precision Nutrition Interventions

After risk stratification, the next step is to design dietary interventions that are both biologically informed and practically achievable [115]. In the Nutri-Exposome Intelligence Framework, intervention design should consider dietary pattern, macronutrient distribution, fiber intake, food processing level, meal timing, micronutrient adequacy, bioactive compounds, cultural dietary preferences, affordability, and long-term adherence [13]. For example, an individual with high glucose variability may benefit from improved carbohydrate quality, increased dietary fiber, and meal-timing adjustment, while an individual with lipid-dominant risk may require targeted modification of saturated fat intake, omega-3 fatty acid intake, and overall dietary fat quality.

Adaptive intervention design is important because dietary response is not fixed [58]. Biological responses may change with weight loss, medication use, microbiome shifts, physical activity, sleep quality, ageing, and disease progression [21]. Therefore, precision nutrition should not be treated as a one-time prescription based only on baseline data. Instead, dietary recommendations should be refined through repeated monitoring of clinical markers, digital phenotypes, dietary adherence, and, where possible, molecular biomarkers.

### 6.3. Building a Feedback-Based Learning System for Nutrition Practice

The final translational step is to convert precision nutrition into a feedback-based learning system [116]. In this model, dietary intervention is followed by continuous or repeated monitoring using tools such as continuous glucose monitoring, wearable activity trackers, sleep monitoring, mobile dietary records, body composition assessment, clinical biomarkers, and selected omics measurements [1]. These feedback data allow researchers and clinicians to evaluate whether the intervention is producing the intended biological effect. For example, improvements in glucose variability, lipid profile, inflammatory markers, liver enzymes, metabolite signatures, or microbiome-derived metabolites may indicate that the intervention is moving the individual toward a healthier metabolic state.

This feedback loop also creates opportunities for n-of-1 nutrition studies and adaptive machine learning models [117]. Instead of assuming that one dietary strategy is optimal for all individuals within a broad disease category, repeated measurements can reveal which intervention works best for a specific person or subgroup over time [43]. As new data are collected, machine learning models can update predictions, refine responder classifications, and support more precise dietary recommendations. In this way, nutri-exposome intelligence transforms precision nutrition from a static recommendation system into a dynamic, data-driven, and biologically responsive model for chronic disease prevention.

## 7. Methodological, Ethical, and Governance Challenges

Although nutri-exposome intelligence offers a promising direction for precision nutrition, its implementation is constrained by several methodological and practical challenges [78]. Data quality, reproducibility, model interpretability, causal validity, privacy, equity, and governance all influence whether such systems can be responsibly translated into research, clinical, or public health settings [38].

### 7.1. Data Quality, Standardization, and Reproducibility

A major challenge in nutri-exposome intelligence is ensuring that input data are reliable, comparable, and reproducible across studies [38]. Dietary intake data remain vulnerable to recall bias, underreporting, portion-size error, and short measurement windows that may not reflect habitual intake [118]. Multi-omics and microbiome datasets introduce additional sources of variability because analytical platforms, sample processing, normalization methods, detection limits, and batch effects can differ substantially between studies. Digital phenotyping tools, including wearable devices and mobile applications, also vary in measurement accuracy, sampling frequency, user adherence, and interoperability.

Future nutri-exposome studies should therefore prioritize standardized reporting and metadata structures across dietary assessment, food processing level, meal timing, supplement use, medication exposure, physical activity, sleep, microbiome sampling, omics processing, and clinical phenotyping [119]. Analytical workflows should document preprocessing decisions, missing-data handling, batch-effect correction, model development, external validation, and sensitivity analyses in a transparent manner [38]. Such standardization is essential not only for improving model reproducibility but also for enabling cross-cohort comparison, meta-analysis, and responsible translation of nutri-exposome intelligence into clinical and public health settings.

### 7.2. Model Interpretability, Causality, and Clinical Validity

Machine learning models can identify complex patterns across dietary, molecular, microbial, clinical, and digital data, but prediction alone is not sufficient for precision nutrition [120]. A model may accurately classify disease risk or dietary response without explaining the biological mechanism behind the prediction [40]. This is problematic in nutrition practice because clinicians, dietitians, researchers, and patients need to understand why a recommendation is being made. For example, a dietary intervention should ideally be linked to interpretable features such as glucose variability, fiber intake, lipidomic profile, inflammatory markers, microbial metabolite production, or genetic susceptibility, rather than being generated from an opaque algorithmic output.

Another challenge is the distinction between correlation and causation [121]. Many nutrition, microbiome, and omics studies are observational, making it difficult to determine whether a biomarker is a cause, consequence, or correlate of disease risk [1]. Machine learning can improve prediction, but it does not automatically establish causal relationships. Therefore, nutri-exposome intelligence should be supported by longitudinal cohorts, randomized dietary interventions, n-of-1 trials, mechanistic studies, causal inference methods, and external validation across independent populations. Clinical validity will depend not only on whether models predict outcomes accurately, but also on whether model-guided interventions improve measurable health outcomes compared with standard dietary care.

### 7.3. Ethics, Equity, and Data Governance

Nutri-exposome intelligence requires the collection and integration of sensitive data, including genetic profiles, dietary behavior, clinical biomarkers, microbiome composition, wearable sensor data, geolocation-linked food environment information, and lifestyle patterns [122]. These data can reveal highly personal information about disease risk, eating behavior, socioeconomic status, and daily routines [38]. Robust data governance is therefore essential to protect privacy, prevent unauthorized use, and ensure that individuals understand how their data will be collected, stored, analyzed, shared, and translated into recommendations. Particular caution is needed when commercial platforms use genetic or digital nutrition data to provide personalized advice that may not yet be supported by strong clinical evidence.

Equity is another central concern [118]. If nutri-exposome models are trained mainly on data from affluent, urban, or European-ancestry populations, their predictions may be less accurate for underrepresented groups [38]. This may widen existing health disparities and limit the usefulness of precision nutrition in diverse public health settings. Future implementation should prioritize multi-ethnic cohorts, culturally relevant dietary databases, affordable testing strategies, transparent algorithms, and governance models that support responsible data sharing. For regions with diverse dietary traditions and rapidly changing chronic disease burdens, including Asian populations, the development of locally relevant nutri-exposome datasets will be especially important for ensuring that precision nutrition is both scientifically valid and socially inclusive.

### 7.4. Practical, Computational, and Equity Barriers to Implementation

Several practical barriers limit the immediate implementation of nutri-exposome intelligence [123]. First, many nutrition datasets are small, relative to the dimensionality of omics, microbiome, wearable, and environmental exposure data [38]. This increases the risk of overfitting, unstable feature selection, and poor generalization across populations. Second, dietary data are affected by recall error, underreporting, day-to-day variation, and inconsistent capture of food processing, meal timing, and cultural dietary practices. Third, microbiome and multi-omics datasets are vulnerable to batch effects, platform differences, compositionality, incomplete metadata, and analytical heterogeneity. Fourth, clustering and responder-stratification models may identify subgroups that are sensitive to preprocessing decisions and may not reproduce in independent cohorts. Therefore, model development should include transparent preprocessing, nested cross-validation where appropriate, external validation, calibration assessment, sensitivity analysis, and biologically interpretable reporting.

Cost and infrastructure represent equally important barriers [124]. Comprehensive nutri-exposome studies may require sequencing platforms, metabolomics or proteomics facilities, cold-chain biospecimen handling, wearable devices, continuous glucose monitors, secure data storage, high-performance computing, bioinformatics expertise, and multidisciplinary clinical support [38]. These requirements may limit implementation in low-resource settings and may widen health disparities if precision nutrition tools are developed mainly for affluent or highly digitized populations. A tiered implementation strategy is therefore needed, beginning with affordable clinical biomarkers and validated dietary assessment tools before expanding to microbiome, omics, and digital phenotyping where feasible. Privacy-preserving analytics, interoperable data standards, transparent algorithms, and culturally relevant dietary databases will be essential for equitable translation.

## 8. Future Directions and Research Priorities

The next stage of nutri-exposome intelligence will require stronger evidence, more diverse datasets, and clearer pathways for clinical and public health translation [61]. Longitudinal cohorts, causal validation, interpretable models, adaptive feedback systems, and equitable implementation will be central to moving the field beyond conceptual integration [60].

### 8.1. Building Longitudinal and Multi-Ethnic Nutri-Exposome Cohorts

The next phase of nutri-exposome research should prioritize large-scale longitudinal cohorts that integrate dietary intake, environmental exposure, clinical phenotypes, microbiome profiles, multi-omics data, and digital health monitoring over time [74]. Cross-sectional studies can identify associations, but they are limited in their ability to capture temporal relationships between dietary exposure, molecular response, and chronic disease development [98]. Longitudinal designs are especially important because diet-related diseases often emerge gradually through cumulative metabolic, inflammatory, microbial, and regulatory changes. Repeated measurements can help distinguish short-term dietary responses from stable biological signatures that predict long-term disease risk.

Multi-ethnic representation should be a central priority in these cohorts [62]. Many existing precision nutrition and omics datasets are biased toward specific populations, which may reduce the generalizability of predictive models [60]. Genetic background, dietary culture, food preparation practices, microbiome composition, socioeconomic context, and environmental exposures differ substantially across populations. Therefore, future nutri-exposome studies should include diverse ethnic groups, culturally specific dietary databases, regionally relevant food exposure information, and population-specific validation strategies. This is particularly important for Asian populations, where rapid nutrition transition, urbanization, and rising cardiometabolic disease burden create an urgent need for locally relevant precision nutrition models.

### 8.2. Advancing Causal, Explainable, and Clinically Actionable Models

Future work should move beyond predictive modelling toward causal and biologically interpretable nutri-exposome intelligence [125]. Machine learning models may identify high-risk individuals or predict dietary response, but clinical translation requires understanding why a specific prediction is made and whether modifying a dietary exposure will improve health outcomes [40]. This requires stronger integration of causal inference methods, randomized dietary interventions, n-of-1 trials, mechanistic validation, and longitudinal multi-omics analysis. Such approaches can help determine whether specific dietary patterns, microbial metabolites, molecular signatures, or environmental exposures play a causal role in disease progression or intervention response.

Explainable artificial intelligence will also be essential for implementation in nutrition practice [126]. Clinicians, dietitians, researchers, and patients need recommendations that are transparent and biologically meaningful [40]. Future models should therefore be designed to identify interpretable features, such as glucose variability, fiber intake, lipidomic markers, inflammatory proteins, microbiome-derived metabolites, or genetic risk profiles. These features should be linked to practical dietary actions rather than abstract algorithmic outputs. This will allow nutri-exposome intelligence to support clinical decision-making, patient education, and shared dietary planning, rather than remaining a purely computational exercise.

### 8.3. Toward Adaptive and Equitable Precision Nutrition Systems

A major future direction is the development of adaptive precision nutrition systems that learn continuously from individual and population-level data [127]. In such systems, dietary recommendations would not be fixed after baseline assessment [5]. Instead, they would be updated based on longitudinal feedback from continuous glucose monitoring, wearable sensors, dietary records, clinical biomarkers, microbiome profiles, and selected omics measurements. This would allow interventions to be refined according to observed biological response, adherence patterns, lifestyle changes, medication use, and disease progression.

Equity must remain central to this future vision [128]. Advanced precision nutrition systems may increase health disparities if they depend on expensive omics testing, proprietary algorithms, or digital tools that are inaccessible to lower-resource populations [118]. Future research should therefore explore cost-effective biomarkers, scalable dietary assessment tools, privacy-preserving analytics, open and interoperable data standards, and culturally adaptable intervention models. Public health implementation should balance individual-level precision with population-level feasibility, ensuring that nutri-exposome intelligence supports not only personalized health optimization but also equitable chronic disease prevention across diverse communities.

## 9. Conclusions

Precision nutrition is moving beyond generalized dietary advice and isolated gene–diet interactions toward a more integrative understanding of dietary response [129]. The nutri-exposome provides a useful conceptual lens for this transition by framing diet as part of a cumulative exposure–response system shaped by host susceptibility, microbiome function, molecular regulation, lifestyle, environmental context, and longitudinal biological change [45].

In this review, we proposed the Nutri-Exposome Intelligence Framework as a data science-driven model for integrating dietary and environmental exposures, host biological susceptibility, microbiome-mediated biotransformation, multi-omics responses, machine learning-based interpretation, precision intervention, and real-time feedback monitoring [130]. This framework provides a structured pathway for transforming complex nutrition data into predictive, explainable, and adaptive strategies for chronic disease prevention [40].

Successful implementation will require longitudinal and multi-ethnic cohorts, standardized dietary and omics metadata, causal validation, interpretable machine learning, ethical data governance, and equitable access to precision nutrition tools. If these challenges are addressed, nutri-exposome intelligence may help shift nutrition practice toward more dynamic, personalized, and biologically grounded chronic disease prevention.

## Figures and Tables

**Table 1:** Concise conceptual distinctions among precision-nutrition frameworks.

Concept	Core Focus	Framework Contribution	Key Limitation	References
Nutrigenetics	Genetic modifiers of dietary response	Host susceptibility layer	Small effects and population bias	[24,25]
Nutrigenomics	Nutrient effects on gene regulation	Molecular-response layer	Often gene-centred	[26,27]
Exposomics	Cumulative environmental and internal exposures	Life-course exposure context	Broad and difficult to operationalize	[28,29]
Precision nutrition	Individualized dietary strategies	Translational goal	May remain marker-fragmented	[6,30]
Multi-omics nutrition	Integrated molecular profiling	Biological measurement layer	Costly and high-dimensional	[31,32]
Nutri-exposome intelligence	Computational exposure–response–feedback integration	Proposed adaptive framework	Needs validation and feasible implementation	[33,34]

**Table 2:** Core data layers and minimum implementation needs for nutri-exposome studies.

Layer	Minimum Data	Enhanced Data	Main Use	Key Constraint	References
Dietary and environmental exposure	Dietary intake, lifestyle, clinical context	Food processing, timing, pollutants, local food metadata	Define modifiable exposures	Recall bias and incomplete exposure history	[48,49]
Host susceptibility	Age, sex, BMI, family history, baseline health	Genomics, polygenic scores, epigenomics	Interpret individual risk	Population bias and modest effects	[50,51]
Microbiome	16S or selected microbial markers	Shotgun metagenomics, pathways, metabolites	Capture diet-microbe transformation	Batch effects and temporal variability	[52,53]
Multi-omics response	Routine biomarkers and basic metabolite panels	Metabolomics, lipidomics, proteomics, transcriptomics	Link exposure to mechanisms	High cost and dimensionality	[54,55]
Digital and longitudinal feedback	Repeated diet records, activity, sleep	CGM, wearables, n-of-1 monitoring	Track response over time	Participant burden and missing data	[56,57]
Clinical phenotype and integration	Anthropometry, glucose, lipids, liver and renal markers	Repeated trajectories and multimodal integration	Connect profiles to outcomes	Heterogeneity and validation needs	[58,59]

**Table 3:** Representative dataset categories for nutri-exposome intelligence research.

Dataset Category	Examples	Main Data Types	Access Type	Possible Use in Framework	Limitation	References
Public nutrition surveillance datasets	NHANES	Dietary recalls, clinical biomarkers, anthropometry, environmental exposure variables	Public	Exposome-wide association, dietary risk modelling, population-level benchmarking	Cross-sectional design, limited omics and microbiome data	[62,63]
Precision nutrition intervention datasets	Food4Me, PREDICT, personalized nutrition trials	Dietary intake, phenotypes, genotype, microbiome, CGM, postprandial response, intervention outcomes	Often controlled, study-specific, or partially available	Model development, responder stratification, intervention comparison	Access restrictions, population bias, proprietary elements	[64,65]
Large biomedical cohorts	UK Biobank, All of Us-related resources	Genetics, clinical phenotypes, lifestyle, diet, biomarkers, longitudinal health outcomes	Controlled access	Risk prediction, gene–diet interaction, external validation	Dietary data may be less granular than dedicated nutrition studies	[66,67]
Microbiome repositories	ENA, SRA, MG-RAST, curated microbiome datasets	16S rRNA or shotgun metagenomics, metadata	Public or controlled	Microbiome–diet association, microbial feature engineering, external validation	Metadata heterogeneity, compositionality, batch effects	[68,69]
Metabolomics and exposomics repositories	Metabolomics Workbench, Exposome-Explorer-linked resources	Metabolites, exposure biomarkers, pathway annotations	Public or mixed	Biomarker discovery, exposure–response modelling	Variable standardization and incomplete dietary linkage	[70,71]
Digital nutrition and wearable datasets	CGM datasets, mobile food logs, wearable activity and sleep datasets	Time-series glucose, diet logs, activity, sleep, adherence	Often private or controlled	Adaptive modelling, feedback systems, n-of-1 nutrition	Privacy, interoperability, device variability	[72,73]
Prospective nutri-exposome cohorts	Newly designed local or multi-ethnic cohorts	Diet, environment, microbiome, omics, clinical biomarkers, wearables	Investigator-generated	Full framework implementation	High cost, infrastructure needs, participant burden	[61,74]

**Table 4:** Concise machine learning strategies for nutri-exposome integration.

Strategy	Main Role	Typical Inputs	Main Output	Key Caution	References
Data preparation	Harmonize heterogeneous data	Dietary, clinical, omics, microbiome, wearable data	Analysis-ready dataset	Missing data and batch effects	[83,84]
Feature engineering	Create interpretable variables	Diet quality, timing, biomarkers, microbial features	Model-ready features	Feature redundancy	[85,86]
Prediction	Estimate risk or response	Clinical, dietary, omics, microbiome data	Risk or response score	Overfitting and poor validation	[87,88]
Responder stratification	Identify response subgroups	Intervention and longitudinal data	Responder clusters	Unstable clusters	[64,89]
Multi-omics integration	Link exposure to mechanisms	Metabolomics, genomics, microbiome, proteins	Pathways and signatures	High dimensionality	[90,91]
Explainable/adaptive models	Support feedback-based decisions	Repeated biomarkers, CGM, wearable data	Updated recommendation logic	Interpretability and governance	[92,93]

**Table 5:** Concise disease applications of the Nutri-Exposome Intelligence Framework.

Disease Area	Main Nutri-Exposome Focus	Useful Data Layers	Modelling Task	Practical Output	References
Obesity and type 2 diabetes	Diet, glycemic response, microbiome, metabolic flexibility	Dietary records, CGM, biomarkers, microbiome, genomics	Risk and response prediction	Personalized carbohydrate, fibre, timing, and weight strategy	[103,104]
Cardiovascular/CKM risk	Diet, lipids, inflammation, kidney-metabolic interactions	Clinical biomarkers, metabolomics, lifestyle, environmental exposure	Risk stratification	Dietary pattern and cardiometabolic risk reduction plan	[59,105]
MASLD	Dietary excess, liver enzymes, microbiome, lipid metabolism	Diet, liver markers, metabolomics, microbiome, imaging	Subtype and progression prediction	Targeted energy, fat-quality, and metabolic monitoring strategy	[106,107]
Older adults/malnutrition	Diet quality, frailty, inflammation, microbiome, functional decline	Dietary assessment, anthropometry, clinical biomarkers, function	Nutritional risk screening	Personalized protein, energy, and micronutrient support	[108,109]
Low-resource/public health use	Minimum feasible clinical and dietary data	Routine biomarkers, diet tools, anthropometry, local food metadata	Low-cost triage and monitoring	Tiered implementation before advanced omics	[110,111]

## Data Availability

Data sharing is not applicable to this article as no datasets were generated or analyzed during the current study.

## References

[1] LeVatte M., Keshteli A.H., Zarei P., Wishart D.S. (2022). Applications of Metabolomics to Precision Nutrition. Lifestyle Genom..

[2] Wang D.D., Hu F.B. (2018). Precision nutrition for prevention and management of type 2 diabetes. Lancet Diabetes Endocrinol..

[3] Sanders L.M., Allen J.C., Blankenship J., Decker E.A., Christ-Erwin M., Hentges E.J., Jones J.M., Mohamedshah F.Y., Ohlhorst S.D., Ruff J. (2021). Implementing the 2020–2025 Dietary Guidelines for Americans: Recommendations for a path forward. J. Food Sci..

[4] Mozaffarian D. (2016). Dietary and Policy Priorities for Cardiovascular Disease, Diabetes, and Obesity: A Comprehensive Review. Circulation.

[5] Zeevi D., Korem T., Zmora N., Israeli D., Rothschild D., Weinberger A., Ben-Yacov O., Lador D., Avnit-Sagi T., Lotan-Pompan M. (2015). Personalized Nutrition by Prediction of Glycemic Responses. Cell.

[6] de Toro-Martin J., Arsenault B.J., Despres J.P., Vohl M.C. (2017). Precision Nutrition: A Review of Personalized Nutritional Approaches for the Prevention and Management of Metabolic Syndrome. Nutrients.

[7] Ferguson L.R., De Caterina R., Gorman U., Allayee H., Kohlmeier M., Prasad C., Choi M.S., Curi R., de Luis D.A., Gil A. (2016). Guide and Position of the International Society of Nutrigenetics/Nutrigenomics on Personalised Nutrition: Part 1—Fields of Precision Nutrition. J. Nutr. Nutr..

[8] Koromina M., Konstantinidou V., Georgaka M., Innocenti F., Patrinos G.P. (2020). Nutrigenetics and nutrigenomics: Ready for clinical use or still a way to go?. Pers. Med..

[9] Marcum J.A. (2020). Nutrigenetics/Nutrigenomics, Personalized Nutrition, and Precision Healthcare. Curr. Nutr. Rep..

[10] Bordoni L., Gabbianelli R. (2019). Primers on nutrigenetics and nutri(epi)genomics: Origins and development of precision nutrition. Biochimie.

[11] Babu M., Snyder M. (2023). Multi-Omics Profiling for Health. Mol. Cell Proteom..

[12] Heianza Y., Qi L. (2017). Gene-Diet Interaction and Precision Nutrition in Obesity. Int. J. Mol. Sci..

[13] Neveu V., Moussy A., Rouaix H., Wedekind R., Pon A., Knox C., Wishart D.S., Scalbert A. (2017). Exposome-Explorer: A manually-curated database on biomarkers of exposure to dietary and environmental factors. Nucleic Acids Res..

[14] Abeltino A., Hatem D., Serantoni C., Riente A., De Giulio M.M., De Spirito M., De Maio F., Maulucci G. (2024). Unraveling the Gut Microbiota: Implications for Precision Nutrition and Personalized Medicine. Nutrients.

[15] Munzel T., Sorensen M., Hahad O., Nieuwenhuijsen M., Daiber A. (2023). The contribution of the exposome to the burden of cardiovascular disease. Nat. Rev. Cardiol..

[16] Mullins V.A., Bresette W., Johnstone L., Hallmark B., Chilton F.H. (2020). Genomics in Personalized Nutrition: Can You “Eat for Your Genes”?. Nutrients.

[17] Page M.J., McKenzie J.E., Bossuyt P.M., Boutron I., Hoffmann T.C., Mulrow C.D., Shamseer L., Tetzlaff J.M., Akl E.A., Brennan S.E. (2021). The PRISMA 2020 statement: An updated guideline for reporting systematic reviews. Rev. Esp. Cardiol. Engl. Ed..

[18] Page M.J., Moher D., Bossuyt P.M., Boutron I., Hoffmann T.C., Mulrow C.D., Shamseer L., Tetzlaff J.M., Akl E.A., Brennan S.E. (2021). PRISMA 2020 explanation and elaboration: Updated guidance and exemplars for reporting systematic reviews. BMJ.

[19] Neveu V., Nicolas G., Amara A., Salek R.M., Scalbert A. (2023). The human microbial exposome: Expanding the Exposome-Explorer database with gut microbial metabolites. Sci. Rep..

[20] Sejbuk M., Siebieszuk A., Witkowska A.M. (2024). The Role of Gut Microbiome in Sleep Quality and Health: Dietary Strategies for Microbiota Support. Nutrients.

[21] Jardon K.M., Canfora E.E., Goossens G.H., Blaak E.E. (2022). Dietary macronutrients and the gut microbiome: A precision nutrition approach to improve cardiometabolic health. Gut.

[22] Vermeulen R., Schymanski E.L., Barabasi A.L., Miller G.W. (2020). The exposome and health: Where chemistry meets biology. Science.

[23] Asghar W., Khalid N. (2023). Nutrigenetics and nutrigenomics, and precision nutrition. Nutr. Health.

[24] Kiani A.K., Bonetti G., Donato K., Kaftalli J., Herbst K.L., Stuppia L., Fioretti F., Nodari S., Perrone M., Chiurazzi P. (2022). Polymorphisms, diet and nutrigenomics. J. Prev. Med. Hyg..

[25] Livingstone K.M., Celis-Morales C., Navas-Carretero S., San-Cristobal R., Forster H., Woolhead C., O’Donovan C.B., Moschonis G., Manios Y., Traczyk I. (2020). Characteristics of participants who benefit most from personalised nutrition: Findings from the pan-European Food4Me randomised controlled trial. Br. J. Nutr..

[26] Berna G., Oliveras-Lopez M.J., Jurado-Ruiz E., Tejedo J., Bedoya F., Soria B., Martin F. (2014). Nutrigenetics and nutrigenomics insights into diabetes etiopathogenesis. Nutrients.

[27] Corella D., Ordovas J.M. (2018). The role of omics in precision nutrition: Strengths and weaknesses. Nutr. Hosp..

[28] Barouki R., Samson M., Blanc E.B., Colombo M., Zucman-Rossi J., Lazaridis K.N., Miller G.W., Coumoul X. (2023). The exposome and liver disease—How environmental factors affect liver health. J. Hepatol..

[29] Gacesa R., Kurilshikov A., Vich Vila A., Sinha T., Klaassen M.A.Y., Bolte L.A., Andreu-Sanchez S., Chen L., Collij V., Hu S. (2022). Environmental factors shaping the gut microbiome in a Dutch population. Nature.

[30] Ajjan R.A., Battelino T., Cos X., Del Prato S., Philips J.C., Meyer L., Seufert J., Seidu S. (2024). Continuous glucose monitoring for the routine care of type 2 diabetes mellitus. Nat. Rev. Endocrinol..

[31] Plaza-Diaz J., Herrera-Quintana L., Olivares-Arancibia J., Vazquez-Lorente H. (2026). Personalized Nutrition Through the Gut Microbiome in Metabolic Syndrome and Related Comorbidities. Nutrients.

[32] Wang Y., Chen J., Ni Y., Liu Y., Gao X., Tse M.A., Panagiotou G., Xu A. (2024). Exercise-changed gut mycobiome as a potential contributor to metabolic benefits in diabetes prevention: An integrative multi-omics study. Gut Microbes.

[33] Kassem H., Beevi A.A., Basheer S., Lutfi G., Cheikh Ismail L., Papandreou D. (2025). Investigation and Assessment of AI’s Role in Nutrition-An Updated Narrative Review of the Evidence. Nutrients.

[34] Vineis P., Robinson O., Chadeau-Hyam M., Dehghan A., Mudway I., Dagnino S. (2020). What is new in the exposome?. Environ. Int..

[35] Lagoumintzis G., Patrinos G.P. (2023). Triangulating nutrigenomics, metabolomics and microbiomics toward personalized nutrition and healthy living. Hum. Genom..

[36] Kortesniemi M., Noerman S., Karlund A., Raita J., Meuronen T., Koistinen V., Landberg R., Hanhineva K. (2023). Nutritional metabolomics: Recent developments and future needs. Curr. Opin. Chem. Biol..

[37] Beulens J.W.J., Pinho M.G.M., Abreu T.C., den Braver N.R., Lam T.M., Huss A., Vlaanderen J., Sonnenschein T., Siddiqui N.Z., Yuan Z. (2022). Environmental risk factors of type 2 diabetes-an exposome approach. Diabetologia.

[38] Kipnis V., Midthune D., Freedman L., Bingham S., Day N.E., Riboli E., Ferrari P., Carroll R.J. (2002). Bias in dietary-report instruments and its implications for nutritional epidemiology. Public Health Nutr..

[39] Ceballos M.W.G., Sy F.F.A., Akbar A., Taofiq A. (2025). Multi-omics integration in disease research. Prog. Brain Res..

[40] Theodore Armand T.P., Nfor K.A., Kim J.I., Kim H.C. (2024). Applications of Artificial Intelligence, Machine Learning, and Deep Learning in Nutrition: A Systematic Review. Nutrients.

[41] You L., Kou J., Wang M., Ji G., Li X., Su C., Zheng F., Zhang M., Wang Y., Chen T. (2024). An exposome atlas of serum reveals the risk of chronic diseases in the Chinese population. Nat. Commun..

[42] Oesterle I., Pristner M., Berger S., Wang M., Verri Hernandes V., Rompel A., Warth B. (2023). Exposomic Biomonitoring of Polyphenols by Non-Targeted Analysis and Suspect Screening. Anal. Chem..

[43] Sempionatto J.R., Montiel V.R., Vargas E., Teymourian H., Wang J. (2021). Wearable and Mobile Sensors for Personalized Nutrition. ACS Sens..

[44] Nourazarain A., Vaziri Y. (2025). Nutrigenomics meets multi-omics: Integrating genetic, metabolic, and microbiome data for personalized nutrition strategies. Genes Nutr..

[45] Bianchetti G., De Maio F., Abeltino A., Serantoni C., Riente A., Santarelli G., Sanguinetti M., Delogu G., Martinoli R., Barbaresi S. (2023). Unraveling the Gut Microbiome-Diet Connection: Exploring the Impact of Digital Precision and Personalized Nutrition on Microbiota Composition and Host Physiology. Nutrients.

[46] Wild C.P. (2012). The exposome: From concept to utility. Int. J. Epidemiol..

[47] Chang C.W., Hsu J.Y., Su Y.H., Chen Y.C., Hsiao P.Z., Liao P.C. (2023). Monitoring long-term chemical exposome by characterizing the hair metabolome using a high-resolution mass spectrometry-based suspect screening approach. Chemosphere.

[48] Neuhouser M.L., Prentice R.L., Tinker L.F., Lampe J.W. (2023). Enhancing Capacity for Food and Nutrient Intake Assessment in Population Sciences Research. Annu. Rev. Public Health.

[49] Perez Rodrigo C., Aranceta J., Salvador G., Varela-Moreiras G. (2015). Food frequency questionnaires. Nutr. Hosp..

[50] Holzapfel C., Waldenberger M., Lorkowski S., Daniel H., Working Group “Personalized Nutrition” of the German Nutrition Society (2022). Genetics and Epigenetics in Personalized Nutrition: Evidence, Expectations, and Experiences. Mol. Nutr. Food Res..

[51] Odriozola A., Gonzalez A., Alvarez-Herms J., Corbi F. (2024). Host genetics and nutrition. Adv. Genet..

[52] Li C. (2023). Understanding interactions among diet, host and gut microbiota for personalized nutrition. Life Sci..

[53] Perler B.K., Friedman E.S., Wu G.D. (2023). The Role of the Gut Microbiota in the Relationship Between Diet and Human Health. Annu. Rev. Physiol..

[54] Ballanti M., Antonetti L., Mavilio M., Casagrande V., Moscatelli A., Pietrucci D., Teofani A., Interno C., Cardellini M., Paoluzi O. (2024). Decreased circulating IPA levels identify subjects with metabolic comorbidities: A multi-omics study. Pharmacol. Res..

[55] Ramos-Lopez O., Assmann T.S., Astudillo Munoz E.Y., Baquerizo-Sedano L., Barron-Cabrera E., Bernal C.A., Bressan J., Cuevas-Sierra A., Davalos A., De la Cruz-Mosso U. (2025). Guidance and Position of RINN22 regarding Precision Nutrition and Nutriomics. Lifestyle Genom..

[56] Ehrhardt N., Al Zaghal E. (2019). Behavior Modification in Prediabetes and Diabetes: Potential Use of Real-Time Continuous Glucose Monitoring. J. Diabetes Sci. Technol..

[57] Potter T., Vieira R., de Roos B. (2021). Perspective: Application of N-of-1 Methods in Personalized Nutrition Research. Adv. Nutr..

[58] Berry S.E., Valdes A.M., Drew D.A., Asnicar F., Mazidi M., Wolf J., Capdevila J., Hadjigeorgiou G., Davies R., Al Khatib H. (2020). Human postprandial responses to food and potential for precision nutrition. Nat. Med..

[59] Zhou M., Sun W., Gao Y., Jiang B., Sun T., Xu R., Zhang X., Wang Q., Xuan Q., Ma S. (2025). Metabolomic profiling reveals interindividual metabolic variability and its association with cardiovascular-kidney-metabolic syndrome risk. Cardiovasc. Diabetol..

[60] Sud M., Fahy E., Cotter D., Azam K., Vadivelu I., Burant C., Edison A., Fiehn O., Higashi R., Nair K.S. (2016). Metabolomics Workbench: An international repository for metabolomics data and metadata, metabolite standards, protocols, tutorials and training, and analysis tools. Nucleic Acids Res..

[61] Denny J.C., Rutter J.L., Goldstein D.B., Philippakis A., Smoller J.W., Jenkins G., Dishman E., All of Us Research Program Investigators (2019). The "All of Us" Research Program. N. Engl. J. Med..

[62] Johnson C.L., Paulose-Ram R., Ogden C.L., Carroll M.D., Kruszon-Moran D., Dohrmann S.M., Curtin L.R. (2013). National health and nutrition examination survey: Analytic guidelines, 1999–2010. Vital Health Stat..

[63] Parker J.D., Kruszon-Moran D., Mohadjer L.K., Dohrmann S.M., Van de Kerckhove W., Clark J., Burt V.L. (2017). National Health and Nutrition Examination Survey: California and Los Angeles County, Estimation Methods and Analytic Considerations, 1999–2006 and 2007–2014. Vital Health Stat..

[64] Merino J. (2022). Precision nutrition in diabetes: When population-based dietary advice gets personal. Diabetologia.

[65] Metwally A.A., Park H., Wu Y., McLaughlin T., Snyder M.P. (2026). Use of Continuous Glucose Monitoring With Machine Learning to Identify Metabolic Subphenotypes and Inform Precision Lifestyle Changes. J. Diabetes Sci. Technol..

[66] Ling C.W., Zhong H., Zeng F.F., Chen G., Fu Y., Wang C., Zhang Z.Q., Cao W.T., Sun T.Y., Ding D. (2024). Cohort Profile: Guangzhou Nutrition and Health Study (GNHS): A Population-based Multi-omics Study. J. Epidemiol..

[67] Navratilova H.F., Lanham-New S., Whetton A.D., Geifman N. (2024). Associations of Diet with Health Outcomes in the UK Biobank: A Systematic Review. Nutrients.

[68] Katz K., Shutov O., Lapoint R., Kimelman M., Brister J.R., O’Sullivan C. (2022). The Sequence Read Archive: A decade more of explosive growth. Nucleic Acids Res..

[69] Kumar G., Bhadury P. (2024). Dataset of metagenomic profiles of human gut microbiome from frozen fecal samples sequenced using Illumina and ONT chemistries. Data Brief.

[70] Barupal D.K., Fiehn O. (2019). Generating the Blood Exposome Database Using a Comprehensive Text Mining and Database Fusion Approach. Environ. Health Perspect..

[71] Wishart D.S., Guo A., Oler E., Wang F., Anjum A., Peters H., Dizon R., Sayeeda Z., Tian S., Lee B.L. (2022). HMDB 5.0: The Human Metabolome Database for 2022. Nucleic Acids Res..

[72] Abeltino A., Riente A., Bianchetti G., Serantoni C., De Spirito M., Capezzone S., Esposito R., Maulucci G. (2025). Digital applications for diet monitoring, planning, and precision nutrition for citizens and professionals: A state of the art. Nutr. Rev..

[73] Zhou Z., Jin D., He J., Zhou S., Wu J., Wang S., Zhang Y., Feng T. (2024). Digital Health Platform for Improving the Effect of the Active Health Management of Chronic Diseases in the Community: Mixed Methods Exploratory Study. J. Med. Internet Res..

[74] Sudlow C., Gallacher J., Allen N., Beral V., Burton P., Danesh J., Downey P., Elliott P., Green J., Landray M. (2015). UK biobank: An open access resource for identifying the causes of a wide range of complex diseases of middle and old age. PLoS Med..

[75] Ferrario P.G., Gedrich K. (2024). Machine learning and personalized nutrition: A promising liaison?. Eur. J. Clin. Nutr..

[76] Singh V. (2023). Current challenges and future implications of exploiting the omics data into nutrigenetics and nutrigenomics for personalized diagnosis and nutrition-based care. Nutrition.

[77] Mendes-Soares H., Raveh-Sadka T., Azulay S., Ben-Shlomo Y., Cohen Y., Ofek T., Stevens J., Bachrach D., Kashyap P., Segal L. (2019). Model of personalized postprandial glycemic response to food developed for an Israeli cohort predicts responses in Midwestern American individuals. Am. J. Clin. Nutr..

[78] Russo S., Bonassi S. (2022). Prospects and Pitfalls of Machine Learning in Nutritional Epidemiology. Nutrients.

[79] Kirk D., Kok E., Tufano M., Tekinerdogan B., Feskens E.J.M., Camps G. (2022). Machine Learning in Nutrition Research. Adv. Nutr..

[80] Celis-Morales C., Livingstone K.M., Marsaux C.F., Macready A.L., Fallaize R., O’Donovan C.B., Woolhead C., Forster H., Walsh M.C., Navas-Carretero S. (2017). Effect of personalized nutrition on health-related behaviour change: Evidence from the Food4Me European randomized controlled trial. Int. J. Epidemiol..

[81] Khorraminezhad L., Leclercq M., Droit A., Bilodeau J.F., Rudkowska I. (2020). Statistical and Machine-Learning Analyses in Nutritional Genomics Studies. Nutrients.

[82] Capocasa M., Venier D. (2026). Artificial intelligence in nutrition science: Balancing innovation and ethical responsibility. Nutr. Health.

[83] Raajaraam L., Raman K. (2024). Modeling Microbial Communities: Perspective and Challenges. ACS Synth. Biol..

[84] Zheng J., Wang J., Shen J., An R. (2024). Artificial Intelligence Applications to Measure Food and Nutrient Intakes: Scoping Review. J. Med. Internet Res..

[85] Hu G., Ahmed M., L’Abbe M.R. (2023). Natural language processing and machine learning approaches for food categorization and nutrition quality prediction compared with traditional methods. Am. J. Clin. Nutr..

[86] Roy G., Prifti E., Belda E., Zucker J.D. (2024). Deep learning methods in metagenomics: A review. Microb. Genom..

[87] Li Z., Wu W., Kang H. (2024). Machine Learning-Driven Metabolic Syndrome Prediction: An International Cohort Validation Study. Healthcare.

[88] Zhu G., Song Y., Lu Z., Yi Q., Xu R., Xie Y., Geng S., Yang N., Zheng L., Feng X. (2025). Machine learning models for predicting metabolic dysfunction-associated steatotic liver disease prevalence using basic demographic and clinical characteristics. J. Transl. Med..

[89] Celis-Morales C., Livingstone K.M., Petermann-Rocha F., Navas-Carretero S., San-Cristobal R., O’Donovan C.B., Moschonis G., Manios Y., Traczyk I., Drevon C.A. (2019). Frequent Nutritional Feedback, Personalized Advice, and Behavioral Changes: Findings from the European Food4Me Internet-Based RCT. Am. J. Prev. Med..

[90] Li P., Luo H., Ji B., Nielsen J. (2022). Machine learning for data integration in human gut microbiome. Microb. Cell Factories.

[91] Wang R., Li B., Lam S.M., Shui G. (2020). Integration of lipidomics and metabolomics for in-depth understanding of cellular mechanism and disease progression. J. Genet. Genom..

[92] Atwal K. (2024). Artificial intelligence in clinical nutrition and dietetics: A brief overview of current evidence. Nutr. Clin. Pract..

[93] Dai Y., Qian Y., Qu Y., Guan W., Xie J., Wang D., Butler C., Dashper S., Carroll I., Divaris K. (2025). Decoding longitudinal microbiome trajectories: An interpretable machine learning approach for biomarker discovery and prediction. Brief. Bioinform..

[94] Li D., Lin J., Yang H., Zhou L., Li Y., Xu Z., Sun L., Zhang X., Xu W., Wang Y. (2025). Causal association of modifiable factors with cardiometabolic multimorbidity: An exposome-wide Mendelian randomization investigation. Cardiovasc. Diabetol..

[95] Liu X., Xu M., Wang H., Zhu L. (2025). Integrating Precision Medicine and Digital Health in Personalized Weight Management: The Central Role of Nutrition. Nutrients.

[96] Ben-Yacov O., Godneva A., Rein M., Shilo S., Lotan-Pompan M., Weinberger A., Segal E. (2023). Gut microbiome modulates the effects of a personalised postprandial-targeting (PPT) diet on cardiometabolic markers: A diet intervention in pre-diabetes. Gut.

[97] Dallio M., Romeo M., Gravina A.G., Masarone M., Larussa T., Abenavoli L., Persico M., Loguercio C., Federico A. (2021). Nutrigenomics and Nutrigenetics in Metabolic- (Dysfunction) Associated Fatty Liver Disease: Novel Insights and Future Perspectives. Nutrients.

[98] Jurek J.M., Zablocka-Sowinska K., Clavero Mestres H., Reyes Gutierrez L., Camaron J., Auguet T. (2025). The Impact of Dietary Interventions on Metabolic Outcomes in Metabolic Dysfunction-Associated Steatotic Liver Disease (MASLD) and Comorbid Conditions, Including Obesity and Type 2 Diabetes. Nutrients.

[99] Rozera T., Pasolli E., Segata N., Ianiro G. (2025). Machine Learning and Artificial Intelligence in the Multi-Omics Approach to Gut Microbiota. Gastroenterology.

[100] Mazilov S.I., Mikerov A.N., Komleva N.E., Zaikina I.V. (2021). The role of nutrigenetics and nutrigenomics in the prophylaxis of chronic non-communicable diseases. Vopr. Pitan..

[101] Sanz Y., Cryan J.F., Deschasaux-Tanguy M., Elinav E., Lambrecht R., Veiga P. (2025). The gut microbiome connects nutrition and human health. Nat. Rev. Gastroenterol. Hepatol..

[102] Wang S., Song S., Gao J., Wu W., Fu Y., Yuan T., Zhao W. (2025). Dynamic Prediction of Postprandial Glycemic Response and Personalized Dietary Interventions Based on Machine Learning. J. Nutr..

[103] Antwi J. (2023). Precision Nutrition to Improve Risk Factors of Obesity and Type 2 Diabetes. Curr. Nutr. Rep..

[104] Del Carmen Fernandez-Figares Jimenez M. (2025). Postprandial Blood Glucose-Focused Dietary Approaches and Their Limitations. J. Nutr..

[105] Guasch-Ferre M., Wittenbecher C., Palmnas M., Ben-Yacov O., Blaak E.E., Dahm C.C., Fall T., Heitmann B.L., Licht T.R., Lof M. (2025). Precision nutrition for cardiometabolic diseases. Nat. Med..

[106] Miller D.M., McCauley K.F., Dunham-Snary K.J. (2025). Metabolic Dysfunction-Associated Steatotic Liver Disease (MASLD): Mechanisms, Clinical Implications and Therapeutic Advances. Endocrinol. Diabetes Metab..

[107] Mogna-Pelaez P., Riezu-Boj J.I., Milagro F.I., Herrero J.I., Elorz M., Benito-Boillos A., Tobaruela-Resola A.L., Tur J.A., Martinez J.A., Abete I. (2024). Inflammatory markers as diagnostic and precision nutrition tools for metabolic dysfunction-associated steatotic liver disease: Results from the Fatty Liver in Obesity trial. Clin. Nutr..

[108] Riddle E., Munoz N., Clark K., Collins N., Coltman A., Nasrallah L., Nishioka S., Scollard T., Simon J.R., Moloney L. (2024). Prevention and Treatment of Malnutrition in Older Adults Living in Long-Term Care or the Community: An Evidence-Based Nutrition Practice Guideline. J. Acad. Nutr. Diet..

[109] Volkert D., Delzenne N., Demirkan K., Schneider S., Abbasoglu O., Bahat G., Barazzoni R., Bauer J., Cuerda C., de van der Schueren M. (2024). Nutrition for the older adult—Current concepts. Report from an ESPEN symposium. Clin. Nutr..

[110] Fleischhacker S.E., Woteki C.E., Coates P.M., Hubbard V.S., Flaherty G.E., Glickman D.R., Harkin T.R., Kessler D., Li W.W., Loscalzo J. (2020). Strengthening national nutrition research: Rationale and options for a new coordinated federal research effort and authority. Am. J. Clin. Nutr..

[111] Mehta S., Huey S.L., Fahim S.M., Sinha S., Rajagopalan K., Ahmed T., Knight R., Finkelstein J.L. (2025). Advances in artificial intelligence and precision nutrition approaches to improve maternal and child health in low resource settings. Nat. Commun..

[112] Sawicki C., Haslam D., Bhupathiraju S. (2023). Utilising the precision nutrition toolkit in the path towards precision medicine. Proc. Nutr. Soc..

[113] Lamurias A., Jesus S., Neveu V., Salek R.M., Couto F.M. (2021). Information Retrieval Using Machine Learning for Biomarker Curation in the Exposome-Explorer. Front. Res. Metr. Anal..

[114] Varayil J.E., Bielinski S.J., Mundi M.S., Bonnes S.L., Salonen B.R., Hurt R.T. (2025). Artificial Intelligence in Clinical Nutrition: Bridging Data Analytics and Nutritional Care. Curr. Nutr. Rep..

[115] Zhang K., Fu Y., Gou W., Miao Z., Tian Y., Liang Y., Liang X., Shuai M., Xiao C., Wang J. (2025). Quantification of personalized glycemic sensitivity to food and its potential for precision nutrition in a series of n-of-1 trials. Am. J. Clin. Nutr..

[116] Mortazavi B.J., Gutierrez-Osuna R. (2023). A Review of Digital Innovations for Diet Monitoring and Precision Nutrition. J. Diabetes Sci. Technol..

[117] Richardson K.M., Jospe M.R., Bohlen L.C., Crawshaw J., Saleh A.A., Schembre S.M. (2024). The efficacy of using continuous glucose monitoring as a behaviour change tool in populations with and without diabetes: A systematic review and meta-analysis of randomised controlled trials. Int. J. Behav. Nutr. Phys. Act..

[118] Mensah G.A., Brown A.G.M., Pratt C.A. (2020). Nutrition Disparities and Cardiovascular Health. Curr. Atheroscler. Rep..

[119] Bailey R.L. (2021). Overview of dietary assessment methods for measuring intakes of foods, beverages, and dietary supplements in research studies. Curr. Opin. Biotechnol..

[120] Fu Y., Gou W., Zhong H., Tian Y., Zhao H., Liang X., Shuai M., Zhuo L.B., Jiang Z., Tang J. (2025). Diet-gut microbiome interaction and its impact on host blood glucose homeostasis: A series of nutritional n-of-1 trials. EBioMedicine.

[121] Cohen Y., Valdes-Mas R., Elinav E. (2023). The Role of Artificial Intelligence in Deciphering Diet-Disease Relationships: Case Studies. Annu. Rev. Nutr..

[122] Slamet-Loedin I.H., Jenie I.A. (2007). Nutrition: Ethics and social implications. Forum Nutr..

[123] Dhanapal A., Wuni R., Ventura E.F., Chiet T.K., Cheah E.S.G., Loganathan A., Quen P.L., Appukutty M., Noh M.F.M., Givens I. (2022). Implementation of Nutrigenetics and Nutrigenomics Research and Training Activities for Developing Precision Nutrition Strategies in Malaysia. Nutrients.

[124] Gyles C.L., Lenoir-Wijnkoop I., Carlberg J.G., Senanayake V., Gutierrez-Ibarluzea I., Poley M.J., Dubois D., Jones P.J. (2012). Health economics and nutrition: A review of published evidence. Nutr. Rev..

[125] Wan M., Simonin E.M., Johnson M.M., Zhang X., Lin X., Gao P., Patel C.J., Yousuf A., Snyder M.P., Hong X. (2025). Exposomics: A review of methodologies, applications, and future directions in molecular medicine. EMBO Mol. Med..

[126] Singer P., Robinson E., Raphaeli O. (2024). The future of artificial intelligence in clinical nutrition. Curr. Opin. Clin. Nutr. Metab. Care.

[127] Livingstone K.M., Celis-Morales C., Navas-Carretero S., San-Cristobal R., Forster H., Woolhead C., O’Donovan C.B., Moschonis G., Manios Y., Traczyk I. (2021). Personalised nutrition advice reduces intake of discretionary foods and beverages: Findings from the Food4Me randomised controlled trial. Int. J. Behav. Nutr. Phys. Act..

[128] Zhu R., Wang R., He J., Wang L., Chen H., Niu X., Sun Y., Guan Y., Gong Y., Zhang L. (2024). Prevalence of Cardiovascular-Kidney-Metabolic Syndrome Stages by Social Determinants of Health. JAMA Netw. Open.

[129] Pena-Romero A.C., Navas-Carrillo D., Marin F., Orenes-Pinero E. (2018). The future of nutrition: Nutrigenomics and nutrigenetics in obesity and cardiovascular diseases. Crit. Rev. Food Sci. Nutr..

[130] Rahimah S., Tallei T.E., Savitri M., Yamada C., Kim H.J., Choi M., Park M.N., Ophinni Y., Kim B. (2026). Molecular basis of precision nutrition: Food components, microbiome-derived metabolites, and multi-omics modeling. Food Chem..

